# Combined Extraction Processes of Lipid from *Chlorella vulgaris* Microalgae: Microwave Prior to Supercritical Carbon Dioxide Extraction

**DOI:** 10.3390/ijms12129332

**Published:** 2011-12-13

**Authors:** Céline Dejoye, Maryline Abert Vian, Guy Lumia, Christian Bouscarle, Frederic Charton, Farid Chemat

**Affiliations:** 1 University of Avignon and Countries of Vaucluse, INRA, UMR408, F-84000 Avignon, France; E-Mails: celine.dejoye@univ-avignon.fr (C.D.); maryline.vian@univ-avignon.fr (M.A.V.); 2 CEA DEN Marcoule, Laboratory of Supercritical fluids and membranes, F-30207 Bagnols sur Cèze, France; E-Mails: guy.lumia@cea.fr (G.L.); christian.bouscarle@cea.fr (C.B.); frederic.charton@cea.fr (F.C.)

**Keywords:** microalgae, supercritical carbon dioxide, microwave, GC, MS, lipid extraction

## Abstract

Extraction yields and fatty acid profiles from freeze-dried *Chlorella vulgaris* by microwave pretreatment followed by supercritical carbon dioxide (MW-SCCO_2_) extraction were compared with those obtained by supercritical carbon dioxide extraction alone (SCCO_2_). Work performed with pressure range of 20–28 Mpa and temperature interval of 40–70 °C, gave the highest extraction yield (w/w dry weight) at 28 MPa/40 °C. MW-SCCO_2_ allowed to obtain the highest extraction yield (4.73%) compared to SCCO_2_ extraction alone (1.81%). Qualitative and quantitative analyses of microalgae oil showed that palmitic, oleic, linoleic and α-linolenic acid were the most abundant identified fatty acids. Oils obtained by MW-SCCO_2_ extraction had the highest concentrations of fatty acids compared to SCCO_2_ extraction without pretreatment. Native form, and microwave pretreated and untreated microalgae were observed by scanning electronic microscopy (SEM). SEM micrographs of pretreated microalgae present tearing wall agglomerates. After SCCO_2_, microwave pretreated microalgae presented several micro cracks; while native form microalgae wall was slightly damaged.

## 1. Introduction

Microalgae are photosynthetic microorganisms that convert the light energy, water and carbon dioxide into algal biomass [1]. The principle of cultivation of microalgae is the same for bacteria, yeasts or molds; it is only the media composition and photosynthesis aspect that distinguishes them [2]. Microalgae are particularly efficient in converting solar energy. Several species of microalgae are rich in oil; the oil content of some microalgae is around 80% of their dry weight in perfect harvest conditions [3]. This rate is often read in the literature and corresponds to the theoretical upper limit generally encountered in laboratory strains grown under extremely controlled, particularly deficient conditions and likely to place the microalgae in lipids “super-production” and mainly triacylglycerol (lipids reserves stored in macronutrient deficiency situations). Microalgae contain two types of lipids, neutral lipids and polar lipids. Phospholipids and glycolipids are polar lipids; phospholipids are concentrated in the membrane structure and glycolipids are predominant in the membranes of photosynthetic organisms. Neutral lipids are essentially mono, di and triacylglycerol; these lipids are stored in cell organelles such as chloroplasts following a deficiency. Polyunsaturated fatty acids are rarely free in the cell, but are mainly located in lipid reserves (triglycerides). Microalgae used the photosynthesis to fix their carbon dioxide by the enzyme Rubisco [4].

In order to extract these lipids, varied solvents are used. More and more organic solvents used in industries were forbidden because of their toxicity or of the reduction of the ozone layer. In particular, the Volatile Organic Compounds (VOCs), usually composed of carbon and hydrogen, can easily be in gaseous form in the atmosphere reaching the troposphere, and thus reduce the ozone layer. On the other hand, VOCs have direct health effects, even at trace level. The using of supercritical fluids as extraction solvents appears as a promising way of replacement, particularly in the prospect of bio-fuel production to improve environmental impact. The supercritical fluid mostly used is carbon dioxide; it is abundant, incombustible, sluggish chemically, non-toxic for the operators, inexpensive and it presents an accessible critical point (31 °C/7.4 MPa) [5]. The supercritical carbon dioxide can solubilize and extract apolar and organic fatty acids. Manipulating the temperature and pressure above the critical points affects the properties of supercritical carbon dioxide and enhances the ability of the supercritical carbon dioxide to penetrate and extract targeted molecules [6]. Conventional methods used to achieve separation and fractionation of different polyunsaturated fatty acids has several major drawbacks. The solvent extraction, possibly coupled with HPLC chromatography resin involves presence solvent residues in the final product. Several research works have been conducted to study the possibilities of extracting lipid by SCCO_2_ [5,7–10].

To improve the efficiency of lipid extraction, thermal pretreatment can be used. Microwave could be an alternative way thanks to their ability to deeply penetrate through the cell wall structure. Microwave irradiation has been used to extract the oil from the biomass [11]. The rapid heating leads to assure high internal temperature, and pressure gradient acting on the cell wall may enhance mass transfer rates without inducing thermal degradation of lipids [11,12].

The objectives of this study were focused on extraction of lipids through microwave assisted-supercritical carbon dioxide extraction (MW-SCCO_2_), which combines two techniques of microwave pretreatment followed by a supercritical carbon dioxide extraction (SCCO_2_).

## 2. Materials and Methods

### 2.1. Microalgae Cultivation and Harvest

*Chlorella vulgaris* was obtained from Alphabiotech at Asserac (Loire-Atlantique, France) and was collected in April 2010. *C. vulgaris* is a species of microscopic unicellular green alga. Through photosynthesis, it reproduces very quickly in fresh water in regions with lots of sunlight and relatively mild temperatures. The microalgae were incubated in freshwater raceway without nutrient deficiency. The biomass was harvested by filtration and centrifugation, then the wet cell mass was frozen overnight at −70 °C and freeze-dried. The apparatus used for freeze-drying is a Cryotec apparatus. The first step is to freeze microalgae (80% water, after centrifugation) followed by the sublimation of ice. It therefore dries the microalgae under vacuum (around 25 Pa) and at −80 °C: the ice becomes vapor and is recovered. The last step begins when all the ice is sublimated. Microalgae are then dried; the temperature rises spontaneously once all free water has been sublimated. Temperatures between 20 and 70 °C for two to six hours can reduce the residual moisture. The percentage of water in the biomass was measured using moisture AnalyzerFreeze Ohaus MB45. Freeze-drying was performed on a microalgal paste with 80% water. Microalgae used in this study contain 15% db (dry basis) water (after freeze-drying) and only 5% db (dry basis) after microwave pretreatment. The total lipids extraction (membranes and reserves lipids) was performed by the Bligh and Dyer method, which is reference method for marine lipid extraction, and therefore is considered as 100% yield. The microalgae used in this study contain mainly membrane lipids such as phospholipids and glycolipids, with a total yield of 5.68 g per 100 g of dry weight biomass. Indeed, the microalgae that have not undergone deficiency do not contain triacylglycerol (lipids reserves stored in macronutrient deficiency situations).

### 2.2. Treatment Operations

#### 2.2.1. Microwave Pretreatment

The pretreatment by microwave has been performed in a classical microwave oven. The output microwave power is variable up to 1000 W and the frequency of 2450 MHz. The power applied is 800 W. 150 ± 0.1 g of dried microalgae (with 15% water content dry basis) were weighed. Microalgae sample was loaded inside a Pyrex bowl. Then, it was placed into the microwave oven to be treated during 6 cycles, each cycle was a 1 min irradiation stage; temperature could then move between 40 °C and 50 °C. The time duration of each cooling cycle along MW treatment was function of temperature; cooling cycle lasted until a temperature of 25 °C in the sample.

#### 2.2.2. Supercritical Carbon Dioxide Extraction

Supercritical carbon dioxide experiments were performed on an SFE apparatus (made by CEA Engineering team, Bagnols sur Cèze, France) mainly consisting of a thermostatic extractor (1 L internal volume) and three separators operated in series. An operation of SCCO_2_ extraction consisted in introducing a quantity of 150 g ± 0.001 g of dried *C. vulgaris* (15 g H_2_O/100 g dry basis after freeze-drying and 5 g H_2_O/100 g dry basis after microwave pretreatment) into a two filter basket inside the supercritical 1 L reactor. All these operations were achieved at isothermal and isobar conditions at 40–70 °C and 20–28 MPa. The CO_2_ flow rate was 10 kg·h^−1^. The duration of extraction was 9 h, they were performed at least three times and the mean values were reported. Extract were collected during extraction, every 30 min, this allowed us to create kinetic profiles. The obtained extracts were kept in flasks shielded from the light, under atmosphere nitrogen at −20 °C to preserve lipid oxidation [13].

### 2.3. Analytical Procedures and Assessments

#### 2.3.1. Scanning Electron Microscopy

Scanning Electron Microscopy (SEM) experiments were performed using a field emission gun scanning electron microscope (model FEI QUANTA 200 ESEM FEG, Hillsboro, OR, USA) equipped with a 1500 °C hot stage. The fragments of microalgae were deposited on an adhesive carbon pastille. The samples were silvered to allow a better conduction of the sample. This metallization was done from a golden thread placed in a tungsten support. A strong decrease of the pressure and an increase of the temperature up to 3000 °C have been realized. The space was broken and the gold was vaporized on the sample [14].

#### 2.3.2. Gas Chromatography Coupled with Mass Spectrometry (GC/MS) Analysis

Extracts were analyzed by GC/MS to identify their fatty acid composition and to compare treatments (SCCO_2_ and MW/SCCO_2_) applied to biomass. These analyses allowed a qualitative and quantitative interpretation between the different treatments. Fatty acids contents of samples were determined using a modified fatty acids methyl ester (FAME) method [15]. A sample (~20 ± 0.01 mg) was weighed into a Pyrex tube fitted with a Teflon-lined cap. Nonadecanoic acid (400 μL of 1 g/L) in dichloromethane was added as internal standard. Boron trifluoride (10%) in methanol (1 mL) was added to methylate the samples, followed by dichloromethane (1 mL) and the mixture was vortex-mixed. The tubes were placed in a heating block of 100 °C for 30 min. Then, the tubes were removed and cooled in room temperature before adding dichloromethane (1 mL) to extracts FAMEs. Hydrogenocarbonate 0.5 M (2 mL) was added, and the mixture was shaken to allow phase separation. The top phase was deleted. This step was realized twice. Anhydrous sodium sulfate was then added to bind any residual water.

Gas-chromatography analysis of the extracts was carried out on GC/MS QP2010 apparatus (Shimadzu, Kyoto, Japan), equipped with split-splitless injector and auto sampler, attached to UBWAX column (30 m × 0.25 mm × 0.50 μm film thickness). Helium was used as carrier gas at a flow rate of 36 cm/s, split ratio 1:20, injector and detector temperature was 250 °C. The oven temperature was programmed at 50 °C during 1 min, then from 50 °C to 150 °C at a rate of 10 °C/min, from 150 °C to 230 °C at a rate of 3 °C/min, 5 min isothermal, then from 230 °C to 240 °C at a rate of 20 °C/min and finally 10 min isothermal. Mass spectra were acquired in EI mode (70 eV); in m/z range 50–360. The amount of 1 μL of sample solution in methanol was injected [15]. The components of extracts were identified by comparison of their mass spectra to those from NIST 8 libraries and their response factors calculated by comparing peak areas of known quantities of standard (Supelco) to the internal standard, nonadecanoic acid.

## 3. Results and Discussion

### 3.1. Preliminary Study

**Figure 1:**
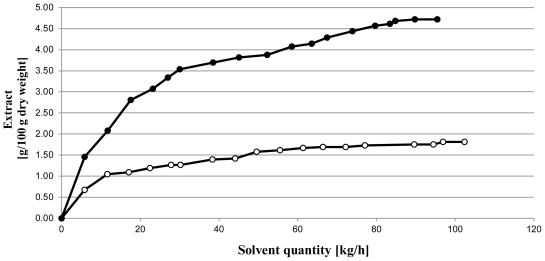
Global extract as a function of extraction time (●) Microwaves/Supercritical carbon dioxide extraction 28 MPa/40 °C; (○) Supercritical carbon dioxide extraction 28 MPa/40 °C.

Lipids of microalgae are contained mainly in the chloroplast and endoplasmic reticulum. The mechanic pretreatment of microalgae before lipid extraction is a method little used; most often the solvent extraction is performed without any pretreatment step. Mendes and Cooney [10,16] explain the pretreatment notion. It was interesting to see the action of a pretreatment on the microalgae’s cell wall. SCCO_2_ extraction was performed in order to compare the impact of microwave pretreatment microalgae *versus* the untreated samples, using two different conditions 28 MPa/70 °C and 28 MPa/40 °C (Table 1). Extraction with microwave pretreated microalgae systematically presented higher yields. This trend was observed till 9 h extraction. The maximum extraction yield could reach 4.73% instead of 1.81% w/w dry weight, and 4.86% instead of 3.90% w/w dry weight, respectively. Uquiche *et al*. [11] and Cooney *et al*. [16] observed a similar increase of extraction yield after a pretreatment of biomass (Table 1). At extraction condition 28 MPa/40 °C, which can be considered as the best in terms of energy saving, the influence of microwave pretreatment was dramatically higher (Figure 1), and could reach 2.61%.

It is important to underline that, the SCCO_2_ pressure increases with the extraction yield (Table 1). Thus, the yield obtained was 4.86% at 28 MPa compared to 2.20% dry basis at 20 MPa for the same SCCO_2_ temperature (70 °C). However, the temperature has simply not enough impact on the differences on yields between 40 °C and 70 °C.

### 3.2. Fatty Acid Composition

The major fatty acid composition of the microalgae was determined using a GC analysis; results are listed in Table 2. Palmitic (C16:0), oleic (C18:1), linoleic (C18:2) and α-linolenic (C18:3) acid are present the highest amounts in good agreement with works found in literature for the fatty acids composition of *C. vulgaris* [17]. The MW pretreatment positively affects these fatty acid quantities of the oil (Table 2). The saturated and polyunsaturated fatty acids were the most abundant in the two methods of MW/SCCO_2_ and SCCO_2_ (with about 71%); while, the main fatty acids, which are palmitic (27% *vs.* 23%), oleic (27% *vs.* 26%), linoleic (10% *vs.* 10%) and α-linolenic acids (16% *vs.* 13%) were present in MW/SCCO_2_ in higher amount than SCCO_2_ (with approximately 80% *vs.* 71% of the total composition). Based on this, one can conclude that MW/SCCO_2_, compared to SCCO_2_, provides the highest concentration of lipids in the extract. This should be due to the impact of the microwave heating on biomass structure.

### 3.3. Scanning Electron Microscopy

**Figure 2:**
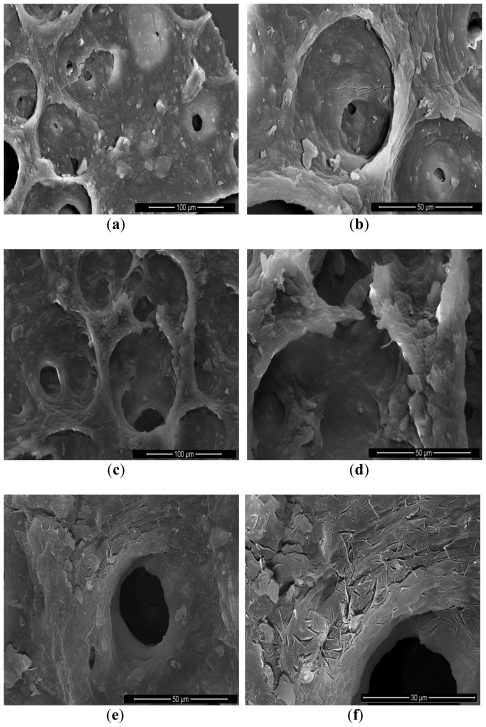
(**a**,**b**) Scanning electronic microscopy SEM scan at 30 kV of native microalgae *Chlorella vulgaris*, magnification_400 and 1000 on original picture scale, 3.6 mm total picture width; (**c**,**d**) SEM scan at 30 kV of microalgae *Chlorella vulgaris* after microwave pretreatment, magnification_400 and 1000 on original picture scale, 3.1 mm total picture width; (**e**,**f**) SEM scan at 30 kV of microalgae *Chlorella vulgaris* after microwave pretreatment and supercritical carbon dioxide extraction, magnification_400 and 1000 on original picture scale, 3.2 mm total picture width.

Microalgae fragments collected after the different extraction processes were observed by SEM (Figure 2) in order to identify the structural impact of the microwave pretreatment and supercritical carbon dioxide extraction at cellular scale. The most important criterion was the aspect of the wall’s agglomerate of microalgae. As shown in Figure 2a,b, the structure of microalgae’s agglomerate after a freeze-dried presents holes on the wall without destroying the wall structure. Contrarily, the structure of the wall was destroyed by microwave as it is highlighted by Figure 2c; it can be seen tearing-off the layer. Figure 2e,f showed the action of SCCO_2_ coupled with microwave pretreatment different impacts on the wall structure were visible. Several micro-cracks have been created by pre-treatment and extraction.

## 4. Conclusions

One can conclude that the MW-SCCO_2_ extraction gives high quality microalgae oil, rich in fatty acids and especially saturated polyunsaturated fatty acids. The results showed important increasing in extraction yields by using the microwaves as a pretreatment stage (between 25% and 150% of increase depending on the conditions). It was found, through the analysis of SEM images, that the microwaves treatment followed by the supercritical extraction gave rise to several damages in the cell wall. In fact, a degradation and breakage microalgae walls appeared after the microwave pretreatment and extraction SCCO2; a strong mechanical effect implied by the microwave and a solubilization of lipids by SCCO_2_.

## Figures and Tables

**Table 1:** Extraction yield obtained by microwaves/supercritical carbon dioxide (MW/SC-CO2) and SC-CO2.

Operating Conditions	Yield (%)	

	MW/SC-CO_2_^a^	SC-CO_2_^b^
280 atm/70 °C c	4.86 ± 0.15	3.90 ± 0.12
280 atm/40 °C d	4.73 ± 0.14	1.81 ± 0.05
200 atm/70 °C e	2.20 ± 0.07	-

a Microwave pretreatment + Supercritical carbon dioxide extraction;

b Supercritical carbon dioxide extraction;

c CO_2_ density 762.13 kg/m^3^;

d CO_2_ density 896.44 kg/m^3^;

e CO_2_ density 650.00 kg/m^3^.

**Table 2:** Comparison of fatty acid composition (percentage level) of oils from microalgae *Chlorella vulgaris* by supercritical carbon dioxide extraction.

Name a	Fatty Acids b	MW/SCCO_2_ c (mg for 100 mg of oils)	SCCO_2_ d (mg for 100 mg of oils)
Lauric acid	C12:0	0.084 ± 0.05	0.083 ± 0.04
Myristic acid	C14:0	0.797 ± 0.26	0.815 ± 0.20
Pentadecylic acid	C15:0	0.452 ± 0.12	0.454 ± 0.11
Palmitic acid	C16:0	26.598 ± 1.66	23.284 ± 1.71
Palmitoleic acid	C16:1	1.761 ± 0.07	1.779 ± 0.06
Palmitolenic acid	C16:2	1.011 ± 0.11	0.698 ± 0.13
Hiragonic acid	C16:3	5.532 ± 0.48	1.145 ± 0.05
Margaric acid	C17:0	3.943 ± 0.15	3.017 ± 0.14
Stearic acid	C18:0	2.061 ± 0.17	2.211 ± 0.21
Oleic acid	C18:1	27.296 ± 1.84	25.939 ± 1.05
Linoleic acid	C18:2	10.402 ± 0.22	10.013 ± 0.19
Linolenic acid	C18:3	16.163 ± 0.79	12.684 ± 0.64
Moroctic acid	C18:4	2.106 ± 0.22	15.149 ± 0.20
Arachidic acid	C20:0	0.590 ± 0.11	2.239 ± 0.44
Behenic acid	C22:0	1.204 ± 0.04	0.487 ± 0.02
∑ SFAs e		35.729	32.590
∑ MUFAs f		29.057	27.718
∑ PUFAs g		35.214	39.689

a Commom name of fatty acids;

b Formula of fatty acids;

c Microwave Pretreatment + Supercritical carbon dioxide extraction;

d Supercritical carbon dioxide extraction;

e Saturated Fatty acids: C12:0 + C14:0 + C15:0 + C16:0 + C17:0 + C18:0 + C20:0+ C22:0;

f Monounsaturated Fatty Acids: C16:1 + C18:1;

g Polyunsaturated Fatty Acids: C16:2 + C16:3 + C18:2 + C18:3 + C18:4.
